# Supplementary material for: Efficient ultrafast field-driven spin current generation for spintronic terahertz frequency conversion
Source: Nat Commun. 2023 Nov 2;14:7010. doi: 10.1038/s41467-023-42845-8 (PMC10622539; doi:10.1038/s41467-023-42845-8)
Supplement: Supplementary file 1 — Supplementary Information [file 41467_2023_42845_MOESM1_ESM.pdf]

Supplementary Information:

Efficient ultrafast field-driven spin current generation for spintronic terahertz frequency conversion

Igor Ilyakov, Arne Brataas, Thales V. A. G. de Oliveira, Alexey Ponomaryov,

Jan-Christoph Deinert, Olav Hellwig, Jürgen Faßbender, Jürgen Lindner,

Ruslan Salikhov and Sergey Kovalev

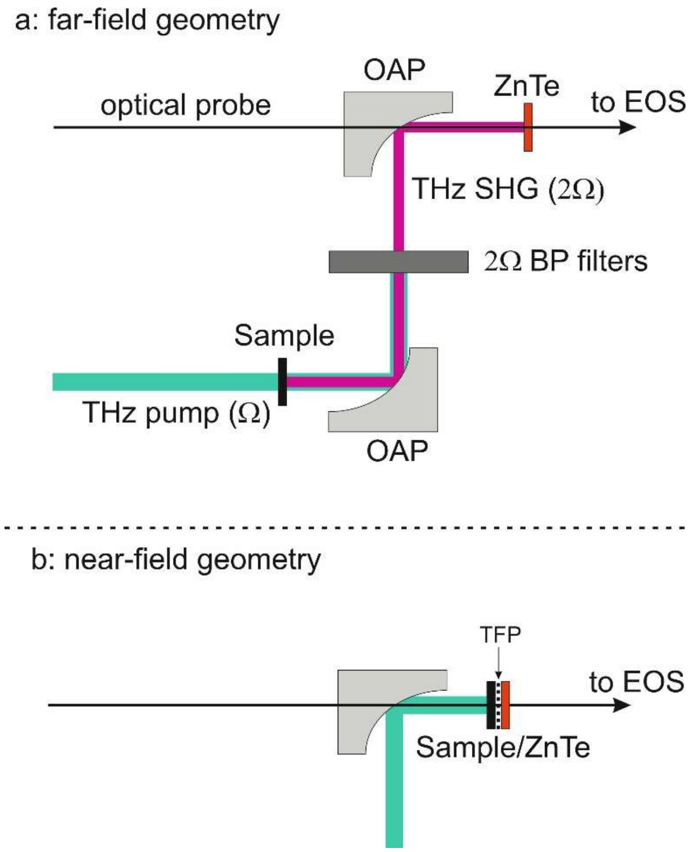

Supplementary Fig. 1 Experimental setup for TSHG and TOR measurements. a) Far-field geometry: The THz pump pulse at 0.5 THz frequency is normally incident onto the sample. The emitted radiation at the second harmonic frequency is separated from the fundamental radiation with the help of two bandpass filters with central frequency at 1 THz, and refocused onto the 2 mm ZnTe crystal for electro-optical sampling. **b)** Near-field geometry: the sample and 100 µm ZnTe are mechanically attached to each other. A thin film wire-grid polarizer (TFP) was inserted between sample and ZnTe crystal. The TFP was oriented for THz transmission polarization orthogonal to the fundamental beam: the fundamental beam is vertically polarized, whereas the TSHG is horizontally polarized. The optical pulse used for EOS transmits through the sample and measures the generated TSHG and TOR fields in the ZnTe. To suppress the fundamental beam

signal in EOS, the ZnTe orientation was tuned to be sensitive only for THz polarization at 90 degrees with respect to the fundamental beam polarization. For far- as well as near-field geometries 35 fs laser pulses at 800 nm central wavelength were used for EOS.

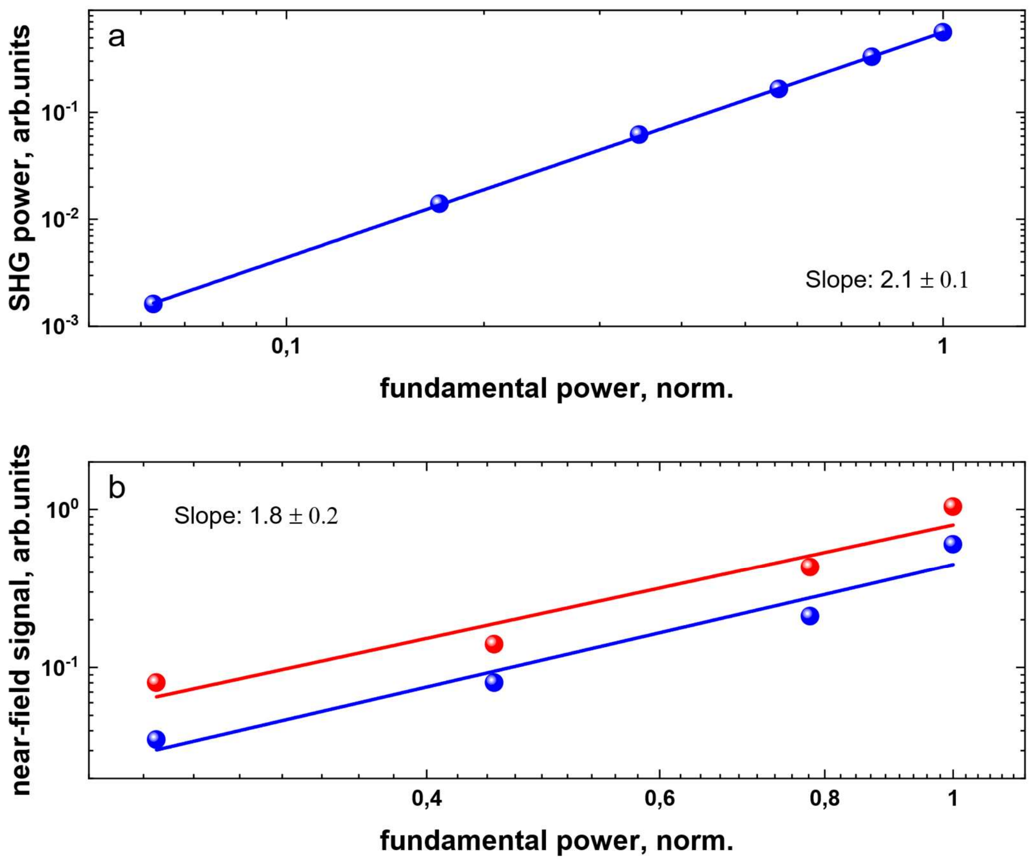

Supplementary Fig. 2 Fluence dependence of THz SHG in Ta/Py/Pt heterostructure. a) TSHG in far field; **b)** near-field TSHG (blue dots) and TOR (red dots)

In Supplementary Figure 2 the dependence of the TSHG and TOR power on the fundamental beam power is shown. The Ta(3nm)/Py(2nm)/Pt(2nm) sample was used at 0.3 THz excitation frequency in the far-field (a), and 0.5 THz excitation frequency in the near-field geometry (b). The sample is vertically magnetized by applying a 50 mT magnetic field, and the measured TSHG and TOR exhibit horizontal polarization for both far- and near-field geometries.

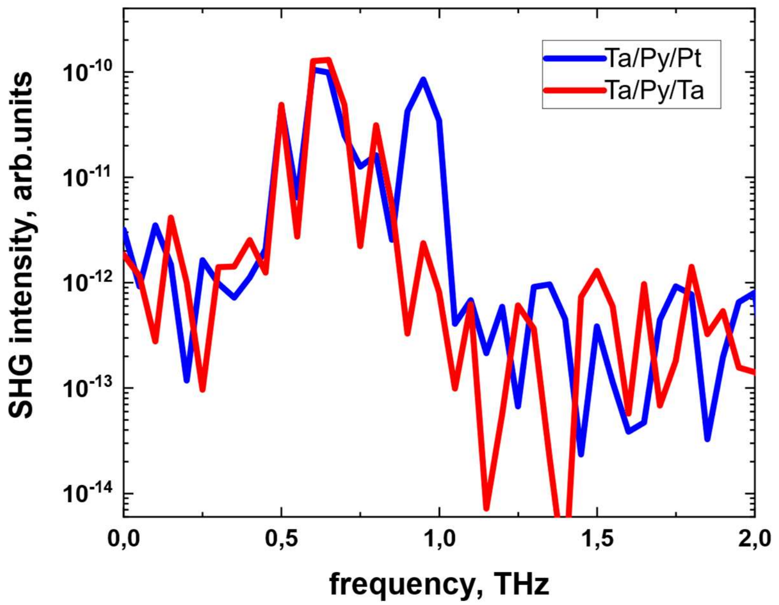

Supplementary Fig. 3 Comparison of the TSHG signals in two different samples with asymmetric Ta(3 nm)/Py(9 nm)/Pt(2 nm) and symmetric Ta(2 nm)/Py(9 nm)/Ta(2 nm) interfaces using 0.5 THz pump. In the symmetric Ta/Py/Ta sample, the TSHG is absent.

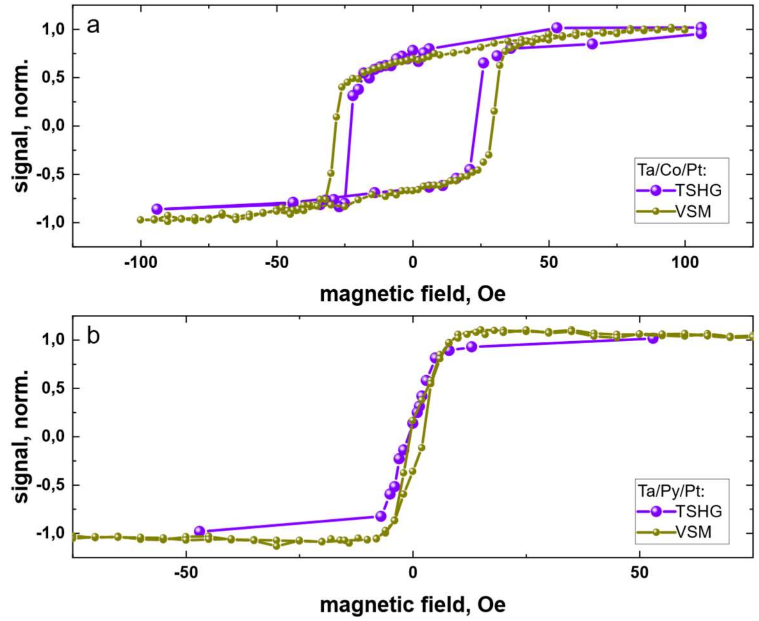

Supplementary Fig. 4 TSHG amplitude measured in Ta(3nm)/Co(2nm)/Pt(2nm) and Ta(3nm)/Py(2nm)/Pt(2nm) samples as a function of the in-plane magnetic field (violet points). The data are compared with vibrating sample magnetometry measurements (green points).

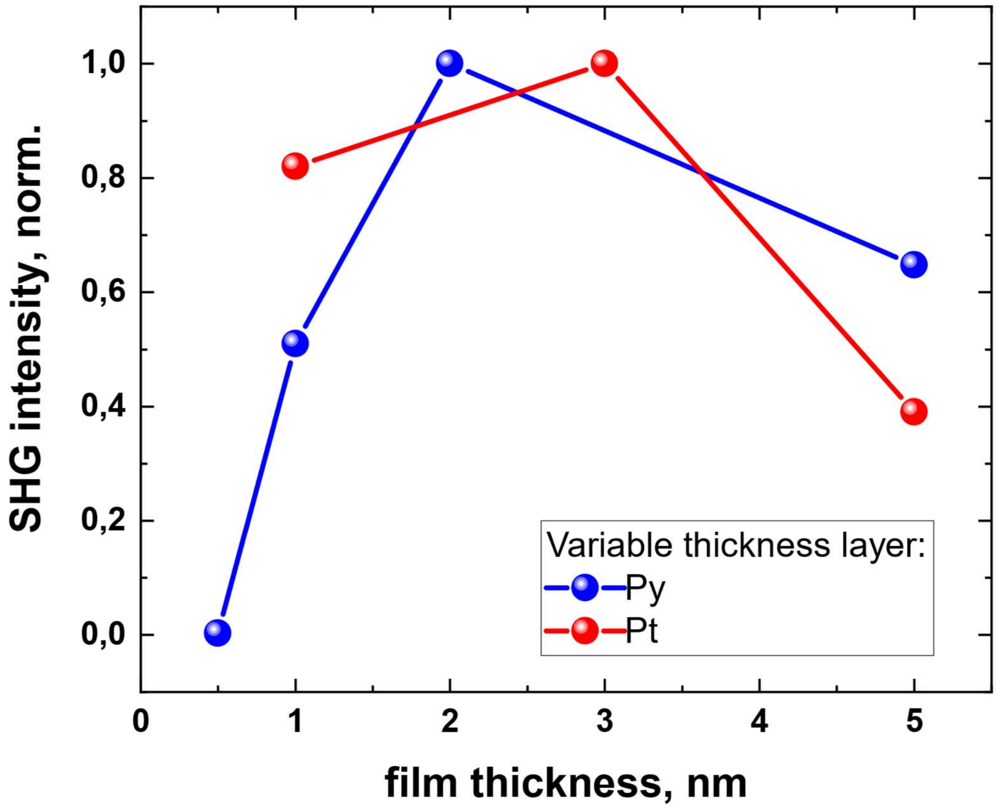

Supplementary Fig. 5 TSHG dependence on Py and Pt layer thickness. Ta(3nm)/Py(X)/Pt(2nm) and Ta(3nm)/Py(2nm)/Pt(X), where X is the variable thickness. Maximal TSHG signal is observed, when Pt and Py layer thicknesses are about 2-3 nm. The optimal thicknesses are similar as in case of STE, and correspond to the spin diffusion lengths in these materials. The TSHG signal is normalized to the sample transmission to account for light screening in metallic materials.

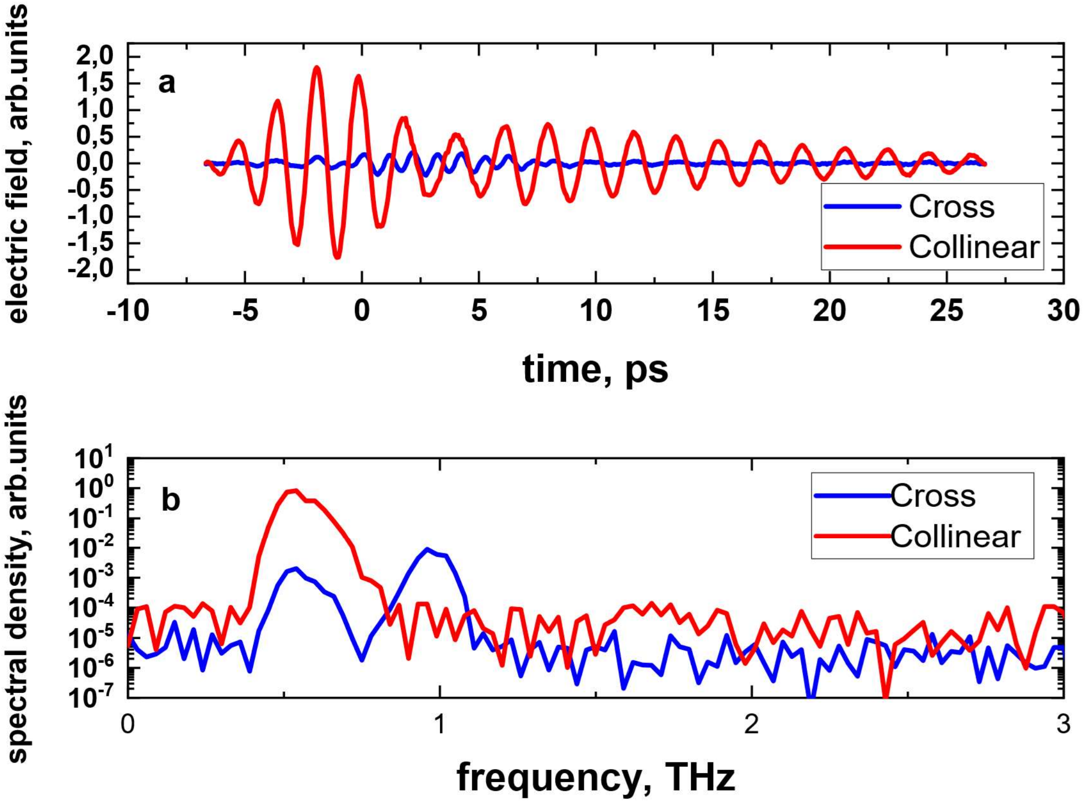

Supplementary Fig. 6. EOS sampling of TSHG (Ta(3nm)/Py(2nm)/Pt(2nm) magnetized vertically) and its fundamental beam leakage measured for collinear (vertical) and cross (horizontal) polarizations. Time-domain measurements (a), and its FFT (b).

To estimate the SHG efficiency, we measured SHG and its fundamental beam leakage after two 1 THz bandpass filters with 20% and 50% bandwidth. The Ta(3nm)/Py(2nm)/Pt(2nm) heterostructure was magnetized vertically leading to horizontal polarization of the SHG. Taking into account the field rejection ratio of two 1 THz filters at 0.5 THz of 2.6×10^{-3}, and the ratio of SHG field to fundamental leakage of 0.1, the SHG field efficiency is about 2.6×10^{-4}. The THz pump spot size on the sample was 800 µm FWHM and it had about 1.5 µJ pulse energy. Using the temporal waveform of the pump beam as shown on Supplementary Fig. 6a, we estimate the fundamental beam peak field strength of about 148 kV/cm, and the corresponding field strength of SHG to be about 39 V/cm.

The effective second order susceptibility $\chi_{eff}^{(2)} = \frac{E_{2\Omega}}{E_{\Omega}^{2}} = \frac{39\,V/cm}{(148\,kV/cm)^2} = 17.8\,pm/V$

Comparison with STE:

In comparison with large-area STEs, the peak power of optical excitation used there was about 8 GW/cm^2 (5.5mJ laser pulses, 40 fs duration, 4.8 cm beam diameter, generated THz pulse energy is about 5.1nJ, Ref. [20]), while in our case it was only 58 MW/cm^2. Considering that STE and TSHG both scale quadratically with pump power, the STE efficiency at 58 MW/cm^2 will be on the order of $8 \times 10^{-9,}$ while the THz SHG efficiency is few times higher 7×10^{-8}.

The STE-based generated THz pulses (5.1nJ, 40 fs, 4.8 cm diameter) corresponds to 7 kW/cm^2.

The conversion between peak power and the electric field can be done via $E = \sqrt{\frac{2*\text{Peakpower}}{c*\varepsilon_0}}$,

In this case the estimated $\chi^{(2)}_{\text{eff}}$ for STE is about 3.8pm/V

Comparison with THz spin-pumping:

In case of MnF_2, a THz magnetic field amplitude of 200 mG acting on the sample has been used, leading to 25 nV voltage that was detected in the adjusted platinum layer with electrodes separated by 1.7 mm, which corresponds to 144 nV/cm of rectified electric field. 200 mG of magnetic field in the electromagnetic wave corresponds to 60 V/cm field strength of the electric component. Taking into account that spin-pumping-based rectification is a second order process, 148 kV/cm driving electric fields (instead of 60 V/cm) will be converted to a rectified field of:

$$V\left(148\frac{kV}{cm}\right) = 144\frac{nV}{cm} * \left(\frac{148\frac{kV}{cm}}{60\frac{V}{cm}}\right)^2 = 0.9\frac{V}{cm}$$

This is about 40 times lower than the 39 V/cm obtained via the spin Seebeck effect in this work.

The effective second order susceptibility $\chi^{(2)}_{\text{eff}} = \frac{E_{\Omega-\Omega}}{E_{\Omega}{}^2} = \frac{144\ \text{nV/cm}}{(60\ \text{V/cm})^2} = 0.4\ \text{pm/V}$

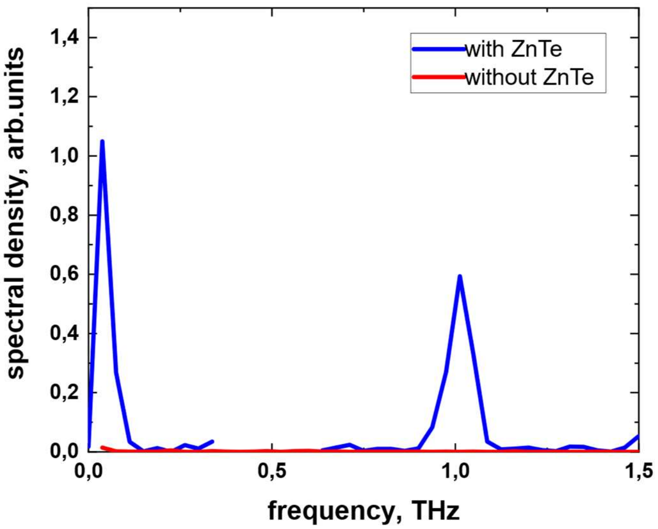

Supplementary Fig. 7 Near-field signals measured with and without ZnTe crystal.

To crosscheck that in case of the near-field geometry we are measuring only the THz-induced electric fields at the surface of the sample, and that we are not sensitive to magneto-optic effects, we have performed measurements with and without ZnTe crystal. In the absence of the ZnTe crystal, the near-field signal goes to zero indicating a negligible contribution to the signal from magneto-optic effects.

Note 1: Pyrospintonic effect:

To estimate the spin accumulation induced by laser heating we used the theoretical description derived by R. Rouzegar et al.[42] According to reference[42], the pyrospintronic spin accumulation obeys the following expression:

$$\mu_s = \gamma \Delta \tilde{T}_{e0} \theta(t) \left[\frac{\Gamma_{es} - \mathrm{R}\Gamma_{ep}}{\Gamma_{es} - \Gamma_{ep}} e^{-\Gamma_{es} t} - \frac{(1-R)\Gamma_{ep}}{\Gamma_{es} - \Gamma_{ep}} e^{-\Gamma_{ep} t} \right], \tag{1}$$

where $\gamma = \frac{\pi^2 k_B^2 T_0}{3} \left(\frac{\partial_\epsilon D_\uparrow}{D_\uparrow} - \frac{\partial_\epsilon D_\downarrow}{D_\downarrow} \right)$, with k_B the Boltzmann constant, T_0 the sample temperature before the laser excitation, $\Delta \tilde{T}_{e0}$ the laser-induced increase of the temperature, Γ_{es}, Γ_{ep} the electron-spin and electron-phonon equilibration rates.

The instantaneous density of states is:

$$D_\sigma = \sum_k \delta\big(\epsilon - \epsilon_{k\sigma}(t)\big), \tag{2}$$

Here we use a simplified quadratic dispersion:

$$\epsilon_{k\sigma} = \frac{\hbar^2 k^2}{2m} - \sigma \frac{\Delta}{2} \tag{3}$$

Δ - is the exchange splitting.

In 3D metals, the density of states is then

$$D_\sigma \propto (\epsilon + \sigma\Delta)^{1/2} \,. \tag{4}$$

Hence

$$\frac{\partial_\epsilon D_\sigma}{D_\sigma} = \frac{1}{2}\frac{1}{\epsilon+\sigma\Delta}, \tag{5}$$

At the Fermi energy, we have

$$\frac{\partial_\epsilon D_\uparrow}{D_\uparrow} - \frac{\partial_\epsilon D_\downarrow}{D_\downarrow} = -\frac{1}{2}\frac{\Delta}{\epsilon^2-\Delta^2} \tag{6}$$

For a strong ferromagnet, Δ is of the order of the Fermi energy ϵ_F, for instance, we assume $\Delta = {}^{\epsilon_F}/_2$

This implies

$$the\ \mu_s \approx \frac{k_B T_0}{\epsilon_F} k_B \Delta \tilde{T}_{e0} \tag{7}$$

The pyrospintronic current is

$$J_s = G\mu_s/e = G\frac{k_B T_0}{e\epsilon_F} k_B \Delta \tilde{T}_{e0}, \tag{8}$$

Where G corresponds to conductance, e – elementary charge. The pyrospintronic current is thus linearly proportional to the sample temperature T_0.

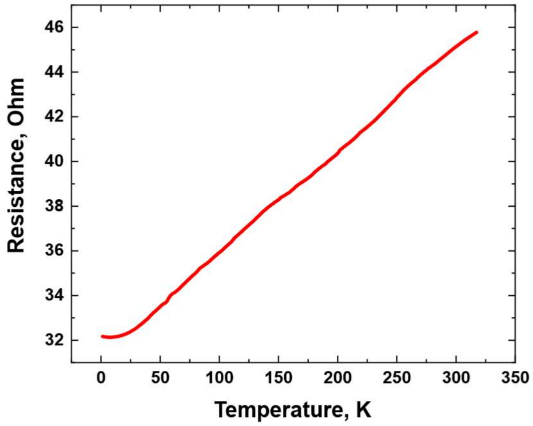

Supplementary Fig. 8 Temperature dependence of 4 nm thick Pt sample resistivity

The resistivity of 4 nm Pt increases about 1.44 times with temperature (liquid helium to room temperature range). Such an increase should result in a twofold increase in THz SHG. The observed increase in THz SHG with temperature was measured to be about 1.6 times. Such a difference can be attributed to the difference in conductivity in 2 nm Pt grown on top of 2 nm Py layer with respect to 4 nm signal Pt layer.
